# The effect of resveratrol, curcumin and quercetin combination on immuno-suppression of tumor microenvironment for breast tumor-bearing mice

**DOI:** 10.1038/s41598-023-39279-z

**Published:** 2023-08-16

**Authors:** Chenchen Li, Yajun Xu, Junfeng Zhang, Yuxi Zhang, Wen He, Jiale Ju, Yinghua Wu, Yanli Wang

**Affiliations:** 1 School of Medicine and School of Environmental and Chemical Engineering, Shanghai University, Shanghai, 200444 People’s Republic of China; 2 Key Laboratory of Tropical Translational Medicine of Ministry of Education, International Associated Research Center for Intelligent Human Computer Collaboration on Tumor Precision Medicine, School of Pharmacy and The First Affiliated Hospital, Hainan Medical University, Haikou, 571199 Hainan China

**Keywords:** Cancer, Immunology, Oncology

## Abstract

Resveratrol, curcumin, and quercetin are the secondary metabolites from medicinal food homology plants, that have been proven their potency in cancer treatment. However, the antitumor effect of a single component is weak. So, herein, we designed an antitumor compound named RCQ composed of resveratrol, curcumin, and quercetin. This study examined the effect on tumorigenesis and development of 4T1 breast cancer-bearing mice following administering RCQ by intragastric administration. RCQ increased the recruitment of T cells and reduced the accumulation of neutrophils and macrophages in the tumor microenvironment. Meanwhile, RCQ suppressed the development of tumor-infiltrating lymphocytes into immunosuppressive cell subpopulations, including CD4^+^ T cells to T helper Type 2 type (Th2), tumor-associated neutrophils (TANs) to the N2 TANs, and tumor-associated macrophages (TAMs) cells to M2 TAMs. RCQ reversed the predominance of immunosuppressive infiltrating cells in the tumor microenvironment and tipped the immune balance toward an immune activation state. In vitro the study showed that RCQ significantly increased reactive oxygen species (ROS), reduce mitochondrial membrane potentials in cancer cells, and modulate pro-apoptotic Bcl-2 family members. In conclusion, RCQ can promote the ROS apoptosis mechanism of tumor cells and alleviate immunosuppression of the tumor microenvironment to enhance the anti-tumor effect.

## Introduction

Compared with synthetic biomaterials, natural edible antitumor polyphenols and flavonoids for medical applications, due to their anti-cancer functions, low side effect, and easy availability, etc., have attracted considerable interest from scientists working in many fields^1–3^. Resveratrol (Res), curcumin (Cur), and quercetin (Que) are known to induce apoptosis in various cancer cells to regulate cancer immunology and cardiovascular disease^4–6^. Resveratrol has been proven that have chemo preventive and chemo therapeutic effects on cancer by acting on NF-κB (nuclear factor kappa-B), Wnt, and PI3K (Phosphatidylinositol 3-kinase)/Akt/mTOR(mammalian target of rapamycin, mTOR), among other pathways, making it a promising anticancer agent^7,8^. And the clinical trials that have been conducted have shown that resveratrol has multiple targets in cells^9^. Curcumin has anticancer effects on different types of cancer by modulating many molecular targets^10,11^. New bioavailable forms of curcumin have been developed, and the results of clinical trials in cancer patients suggest that these drugs may represent promising new therapies for cancer^12^. Quercetin could increase the expression level of pro-apoptotic protein and decrease the expression level of anti-apoptotic protein^13^ and inhibit PI3K/AKT/mTOR and STAT3 (Signal Transducer and Activator of Transcription 3) pathways in cancer cells, thereby down-regulating the expression of survival cell proteins such as Cellular FLICE (FADD-like IL-1β-converting enzyme)-inhibitory protein (c-FLIP), cyclin D1 (cyclin-dependent kinases) and cMyc (a nuclear transcription factor)^14,15^. With the development of clinical trials, quercetin's great potential for cancer treatment has been further confirmed^16^. Therefore, these natural polyphenols represent the effective homology of medicines and foods.

Breast cancer is the most common cancer globally and a leading cause of death among women, posing a general health risk to women^17,18^. Due to the numerous disadvantages of traditional drug treatment for breast cancer, such as weight loss, noticeable side effects, increasing drug resistance, etc., some studies have selected the use of the combination of natural polyphenols and drugs. Studies have shown that dietary supplements can help treat cancer^19^. For example, Cho et al. studied that resveratrol, curcumin, and quercetin could assist with gemcitabine to treat bladder cancer synergistically^20^. Liu et al*.* demonstrated that the combination of curcumin and resveratrol could effectively regulate the occurrence of lung cancer in rats^21^. Gavrilas et al*.* also demonstrated that resveratrol combined with curcumin can treat colon cancer^22^. Fatease et al*.* found that resveratrol, curcumin, and quercetin were found to enhance the efficacy of Adriamycin (ADR) when used alone or in combination^23^. In addition, Patcharawalai Jaisamut et al*.* found that the simultaneous administration of resveratrol and curcumin could enhance the absorption rate of the drug^24^. Chung et al*.* found that flavonoids also enhance the bioavailability of phenols, thereby enhancing the poor bioavailability of the drug themselves^25^. Recent results show that combination therapies can reduce tumor resistance to drugs and side effects while enhancing drug sensitivity^26–28^. In recently published studies, the main research points are drug efficacy, drug combination development, analysis of molecular mechanisms and targets, and transcription factor level research involved in cancer progression^29,30^. In addition, most of these studies mainly evaluate oxidation resistance, natural killer cell activity, and macrophage phagocytosis to judge their ability to prevent disease^31–34^. It is known that high doses of Res have oxidation-promoting properties, and intense ROS production could add to the oxidant action of radiations and induce more damage in the DNA than the cancer cell can repair^35,36^. However, it is still unclear whether Res synergistically enhances the apoptosis of Cur and Que through ROS-mediated endoplasmic reticulum stress and mitochondrial dysfunction. It is worth researching their synergistic effects on the cellular apoptosis process.

Few researchers have observed the use of the dietary supplement on tumor infiltrating lymphocytes (TILs)’ change in tumor immunosuppression. TILs play the leading role in the tumor microenvironment, producing pro-inflammatory factors to attack tumor cells or anti-inflammatory factors to help tumor cells escape and metastasize^37^. TILs, including T lymphocytes, tumor-associated macrophages (TAMs), and Tumor-associated neutrophils (TANs), have different cell subpopulations with positive or negative effects on the tumor microenvironment. According to reports, specific tumor-infiltrating cells, such as CD8^+^ cytotoxic T lymphocytes, type 1 helper CD4^+^ T cells (Th1), M1 TAMs, and N1 TANs cells have anti-tumor activity, while T regulatory (Treg) cells, type 2 helper CD4^+^ T cells (Th2), N2 TANs cells and M2 TAMs known for their immunosuppressive and pro-tumor activity^38,39^. Current studies generally believe that trends of TILs such as CD4^+^T cells and CD8^+^T cells in the tumor microenvironment can predict some tumor conditions^40^. On the other hand, ROS are important signal mediators involved in the activation of T cells and NK cells and are used by neutrophils and macrophages to destroy cancer cells^41,42^.

The efficiency mentioned above has prompted us to use natural polyphenols combinations to fight breast cancer better. Therefore, we designed an antitumor compound named RCQ (a mixture of resveratrol, curcumin, and quercetin) with no side effects and anti-proliferative effects on tumorigenesis and development. It is a way to study whether combination treatment affects the tumor's immune microenvironment. This study's goals were to evaluate the specific curative effect of taking natural polyphenols before and after tumorigenesis and determine the effect of eating natural polyphenols on TILs subpopulations and tumor immunosuppression.

## Results and discussion

### In vitro antitumor effect of RCQ combinations

**Figure 1:**
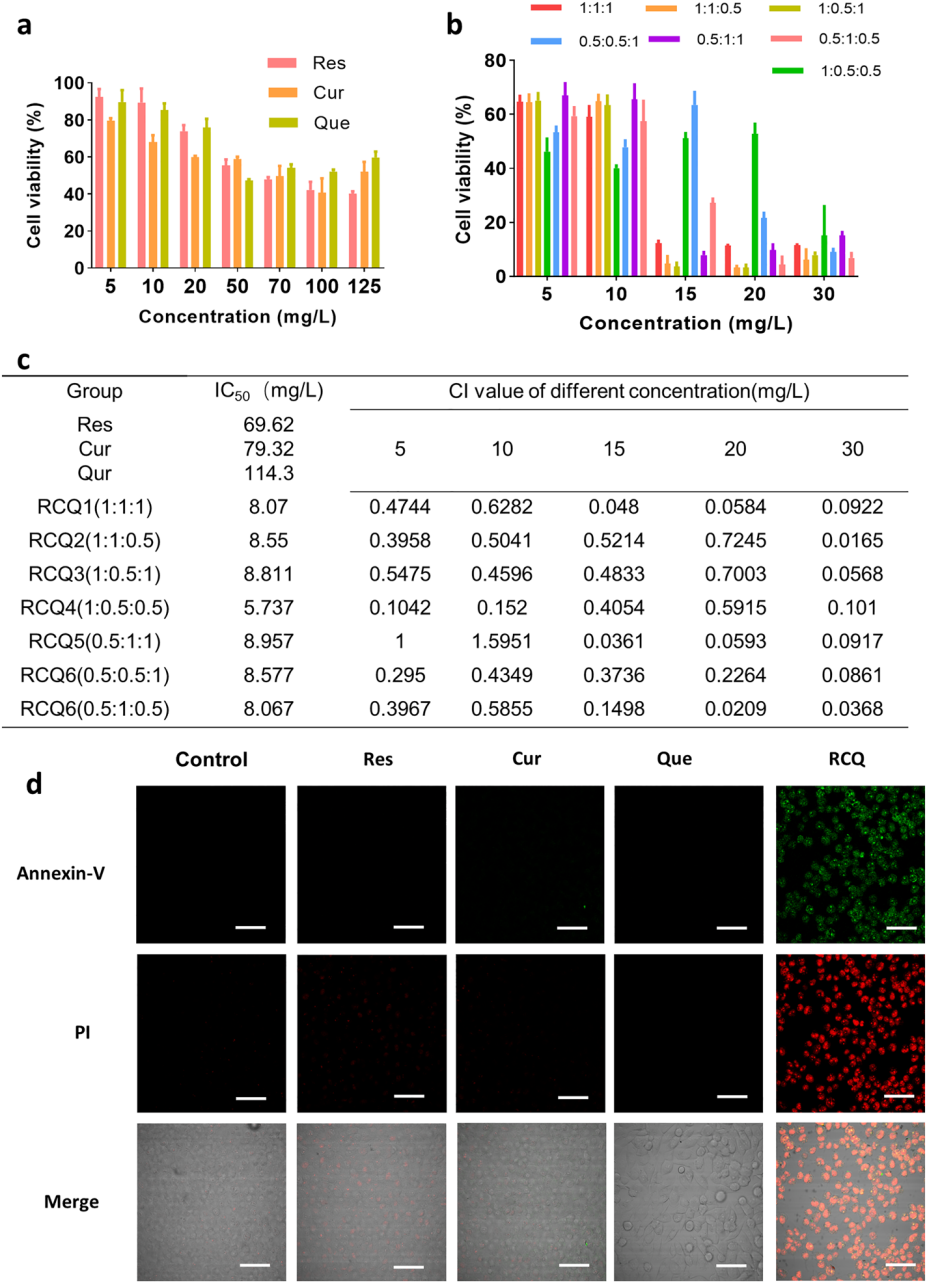
(**a**) 4T1 cell viability of Res/Cur/Que at different concentrations. (**b**) 4T1 cell viability of RCQ with different proportions. (**c**) Combination index (CI) and IC_50_ for different proportions of RCQ. At CI ≤ 1, the components are synergistic. The concentration here referred to the concentration of resveratrol, and other polyphenols were configured in proportion. (**d**) Fluorescence images (40 ×) displayed morphology and Annexin V/PI staining of 4T1 cells after incubation with RCQ 8 h. Error bars represents mean ± SD; scale bar, 100 μm.

As shown in Fig. 1a–c, seven combinations of different ratios were designed to study the best combination for treating cancer. All combinations of different proportions for RCQ had a greater impact on 4T1 cells than Res, Cur, and Que alone. The inhibition rate of RCQ at different combinations was at least nearly 5 times higher than that of a single Res/Cur/Que at the same concentration while the ratio of Res:Cur:Que = 1:1:0.5 was the highest lethality to 4T1 cells and the lowest dose required. As mentioned above, RCQ in the ratio of Res:Cur:Que = 1:1:0.5 had the best antitumor effect. As shown in Fig. 1d by Annexin V and PI staining, RCQ can promote the apoptosis of 4T1 cells more than the low equivalent concentration of natural polyphenols, thus better inhibiting the proliferation of cancer cells. ROS can initiate a chain reaction, which easily reacts with various unsaturated fatty acids and cholesterol on the cell membrane. Such direct oxidative damage to cells can lead to cell apoptosis.

**Figure 2:**
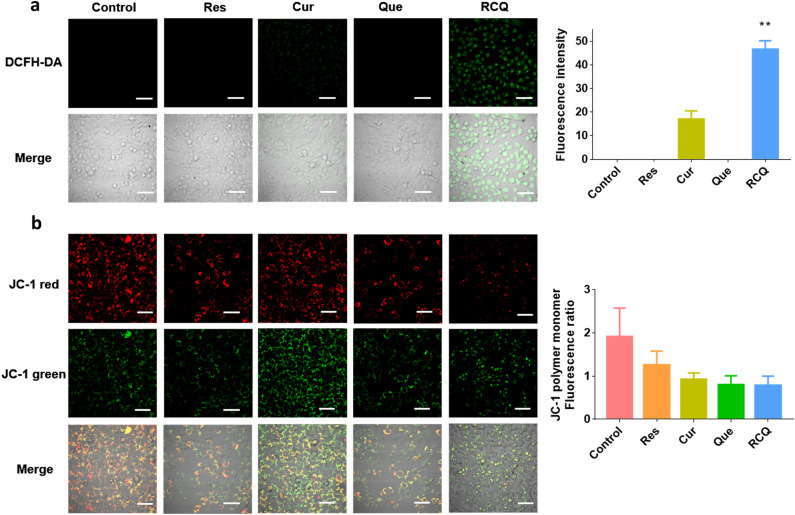
Fluorescence images (40 ×) of (**a**) ROS production in 4T1 cells incubated with 15 mg L^−1^ Res/Cur/Que/RCQ after 2 h. (**b**) Mitochondrial membrane potential (Δ Ψm) visualized by JC-1-staining in 4T1 cells incubated with 15 mg L^−1^ Res/Cur/Que/RCQ after 2 h. scale bar, 100 μm. **p < 0.01.

We detected the ROS production by RCQ in 4T1 cells in Fig. 2a and Fig. S1a, it can be seen that the amount of ROS in 4T1 cells treated with RCQ was higher than that of any single component. The process of cell apoptosis is often accompanied by the destruction of mitochondrial transmembrane potential, which is widely regarded as one of the earliest events in the process of cell apoptosis. In normal cells, when the membrane potential is normal, tetrechloro-tetraethylbenzimidazol carbocyanine iodide (JC-1) enters into the mitochondria through the polarity of the mitochondrial membrane and forms a polymer emitting red fluorescence due to the increased concentration. In apoptotic cells, the mitochondrial transmembrane potential is depolarized, and JC-1 is released from the mitochondria, the concentration decreases, and reverses into a monomer form emitting green fluorescence. Therefore, changes in mitochondrial membrane potential can be detected qualitatively (migration of cell population) and quantitatively (fluorescence intensity of cell population) by detecting green and red fluorescence. In Fig. 2b and Fig. S1b, it can be seen that the Mitochondrial membrane potential (MMP) was decreased after treatment with Res/Cur/Que/RCQ while the RCQ group decreased the most. JC-1 polymer existed in the mitochondria of normal cells, showing strong red fluorescence. After treating with the same concentration of Res, Cur, and Que, the fluorescence intensity decreased to different degrees, but the fluorescence of RCQ decreased most significantly. Obviously, it can be seen the conversion from red fluorescence to green fluorescence. These data indicate that RCQ confers greater damage to the mitochondria in the cells. In other words, the incubation of polyphenols could reduce the mitochondrial membrane potentials of 4T1 cells, but the decline degree of RCQ was the highest, which was consistent with the increase of ROS.

**Figure 3:**
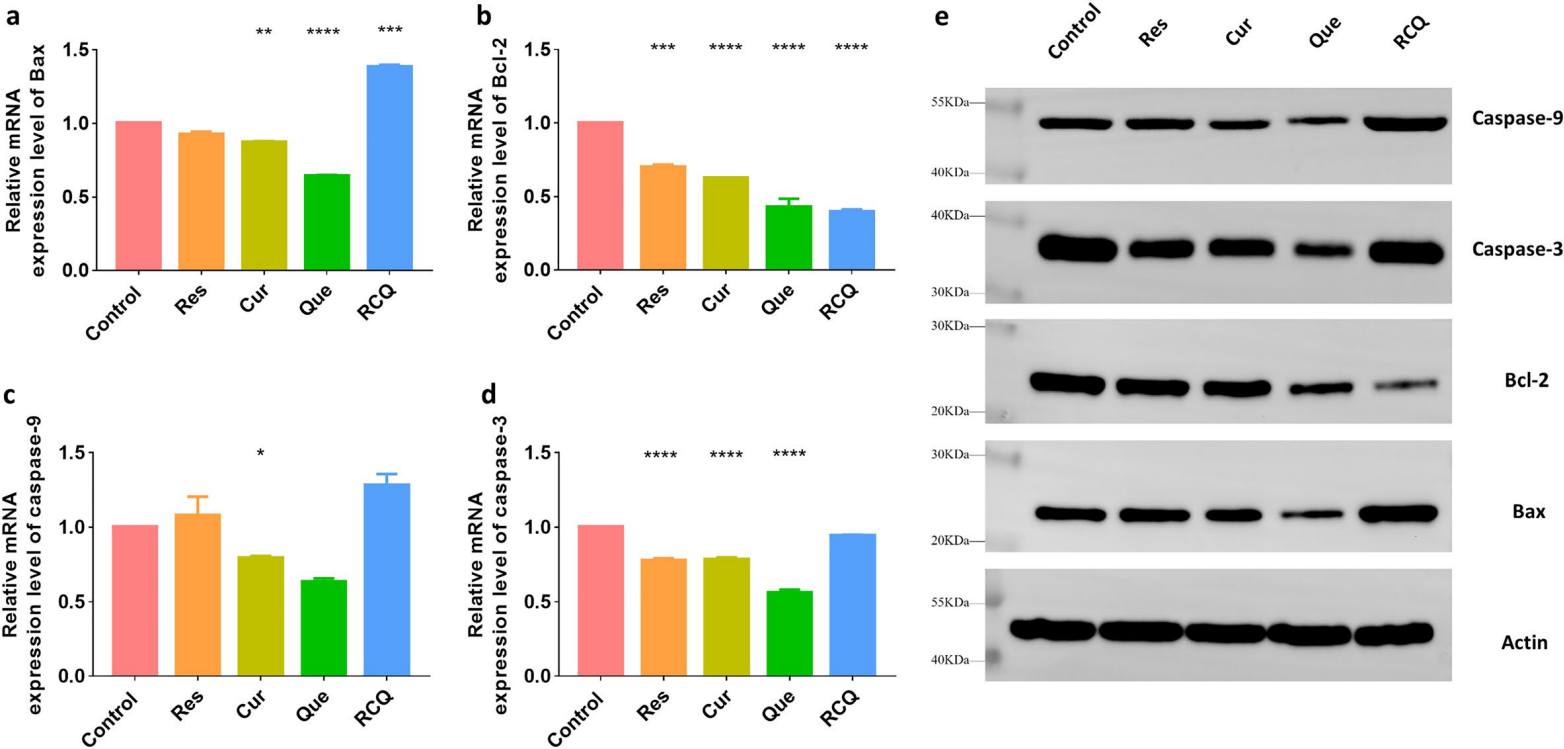
The mRNA expression levels of (**a**) Bax, (**b**) Bcl-2, (**c**) caspase-9, (**d**) caspase-3 in 4T1 cells incubated with 15 mg L^−1^ Res/Cur/Que/RCQ after for 10 h. (**e**) The protein expression levels of Bax/Bcl-2/caspase-9/caspase-3 by Western Blot and the images have been cropped. The original image is in Fig. S4. *p < 0.05, **p < 0.01, ***p < 0.001, ****p < 0.0001 vs control.

To test whether Bcl-2 family members participate in cooperative apoptosis, the mRNA and protein expression levels of Bax, Bcl-2, caspase-3, and caspase-9 were detected by PCR and western blot. (Fig. 3) It can be seen from Fig. 3a that RCQ can promote the production of Bax in 4T1 cells, which was about 1.38 times that of the control group, while other single polyphenols had no significant changes at the same concentration. Similarly, the four groups of drugs can reduce the production of Bcl-2 (Fig. 3b), and the control group is 1.43-fold that of Res, 1.6-fold that of Cur, 2.34-fold that of Que 2.52-fold that of RCQ. In this process, RCQ also increased the level of apoptosis signaling molecule caspase-9, while other polyphenols in the same concentration did not increase significantly. As shown in Fig. 3c, only Res and RCQ increased the production of caspase-9, which were 1.08-fold and 1.28-fold that of the control group, respectively. It had been determined that the caspase-3 activation pathway was independent of mitochondrial cytochrome c release and caspase-9 function, so we also measured the amount of caspase-3 transcription and finally found the relationship with polyphenol incubation was not noticeable (Fig. 3d). It demonstrated that RCQ treatment for 4T1 cells resulted in a decrease in the expression of the anti-apoptotic Bcl-2 protein and an increase in the expression of the pro-apoptotic Bax protein which was also proved by Western Blot (Fig. 3e). The increase of ROS in cells destroyed mitochondrial membrane potential and caused cells to send out apoptosis signals^43^. It can be clearly seen from Fig. 3a,b that the ratio of Bax/Bcl-2 in the RCQ group increases significantly. Apoptotic caspases are functionally subdivided into the initiator caspases (caspase-2, -8, -9, and -10), which are the first to be activated in response to a signal, and the executioner caspases (caspase-3, -6, and -7) that carry out the demolition phase of apoptosis^44,45^. Maybe the 4T1 cells were in the early apoptotic state, the promotion of apoptotic Caspase-3 in cells was not obvious after incubation for 10 h, so the apoptotic caspase-9 was significantly increased. In summary, RCQ can enhance oxidative stress response to promote the loss of mitochondrial membrane potential and increase apoptotic signals.

### In vivo antitumor effect of RCQ combinations

**Figure 4:**
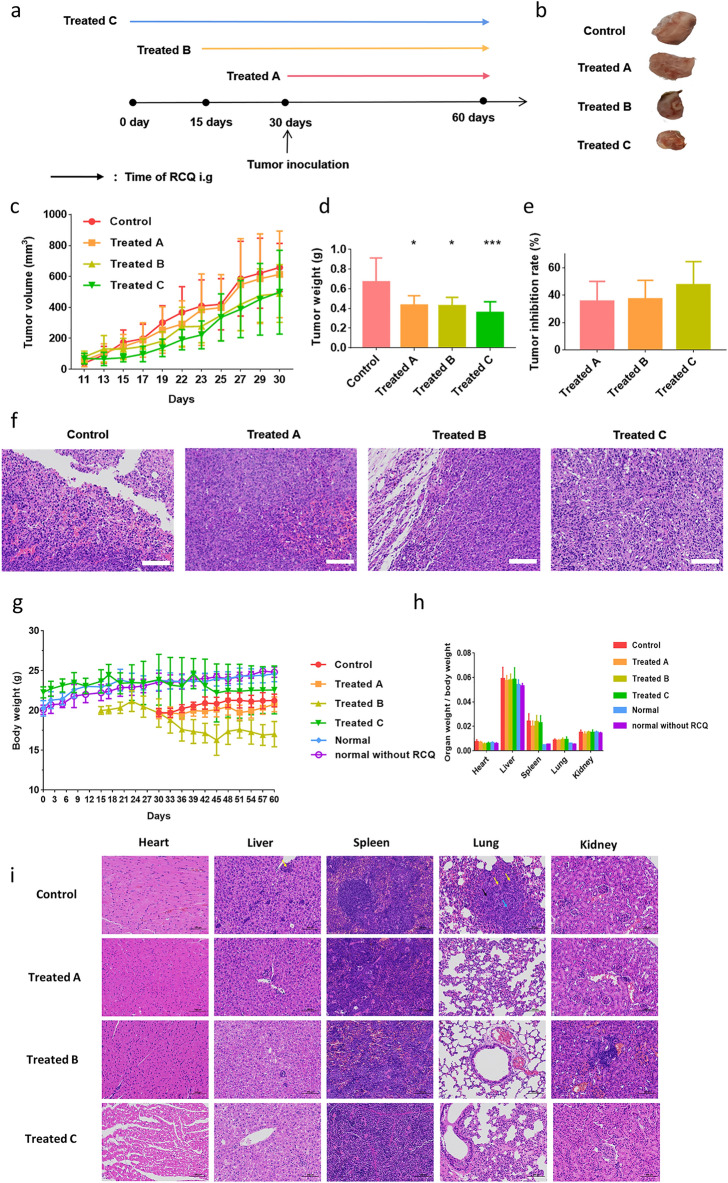
In vivo antitumor effect of RCQ combinations with different administration. (**a**) Flow chart of treatment intervention groups, (**b**) photographs of tumors in mice after dissection, (**c**) tumor volume, (**d**) tumor weight, and (**e**) tumor inhibition rate, (**f**) H&E staining of tumor tissue sections from four groups of mice. scale bar: 100 μm. (**g**) Body weight of mice. (**h**) The organ to body weight ratios of different groups of mice. (**i**) H&E staining of heart, liver, spleen, lung, and kidney. Neutrophil infiltration (yellow arrow), tumor metastasis (black arrow), and blood vessels were replaced by tumor cells (blue arrow), scale bar: 100 μm. Error bars represent mean ± SD. *p < 0.05, **p < 0.01, ***p < 0.001 vs control.

Then, to study the antitumor effect of RCQ on breast cancer-bearing mice, there are four treatment groups were designed as shown in Fig. 4a: (I) The control group was tumor-bearing mice; (II) Treated A group was given RCQ by intragastric administration for 30 days after subcutaneous injection of 4T1 breast cancer cells; (III) Treated B group was consumed RCQ for 15 days for prevention, then injected subcutaneously with 4T1 breast cancer cells and continued to gavage RCQ for 30 days; (IV) Treated C group were consumed RCQ for 30 days for prevention, then injected subcutaneously with 4T1 breast cancer cells and continued to gavage RCQ for 30 days. Normal mice without 4T1 cells were given RCQ intragastric administration for 60 days. Another group of normal mice was not fed RCQ. The body weight of the above mice was recorded.

Mice were sacrificed one day after the last intragastric administration and tumor tissues were collected. From Fig. 4b, the photograph of the tumor showed that RCQ inhibited tumor growth and RCQ inhibited tumor volume growth throughout the whole process (Fig. 4c). The differences in tumor weight between the four groups also demonstrated excellent therapeutic efficiency (Fig. 4d). The tumor growth inhibition rate was calculated based on the tumor weight (Fig. 4e). We could find that the mice that had taken natural polyphenols for a long time (Treated C group) had the most obvious tumor resistance, and the tumor inhibition rate was 47.14%. Followed by the Treated B group, the inhibition rate was 36.02%. At last, the timely intake of RCQ (Treated A group) after cancer could also reduce the growth of tumor volume, with a tumor inhibition rate of 35.36%. Similarly, tumor tissue sections showed that the inflammatory cell infiltration in the tumor microenvironment of mice fed with RCQ was greatly reduced, while a large amount of neutrophil infiltration was seen at the edges of tumor necrosis tissue and local tumor edges in the control group (Fig. 4f). Figure 4g shows that whether RCQ is provided to mice in the tumor group has no discernible impact on their viscera indices or body weight. Because of spleen edema and immune system stress, it should be emphasized that animals with tumors had bigger spleens than normal mice. Degeneration and hypertrophy of the spleen are thought to be caused by inflammation. Figure 4h showed the ratio of the organ to body weight of the mice after being sacrificed and RCQ had no effect on the organ weight of the mice. As can be seen from Fig. 4i, taking RCQ had no effect on the heart and kidney of the tumor-bearing mice, while it could significantly reduce inflammatory cell infiltration in the liver and lungs of the tumor mice, reduce white pulp injury in the spleen, and prevent tumor cell metastasis to the lungs. In conclusion, RCQ can reduce inflammation and inhibit tumor growth in mice.

### Antitumor mechanism of RCQ combinations for breast cancer-bearing mice

#### RCQ increased the recruitment of T cells in tumor microenvironment and transformed CD4^+^ T cells to Th1 cells

**Figure 5:**
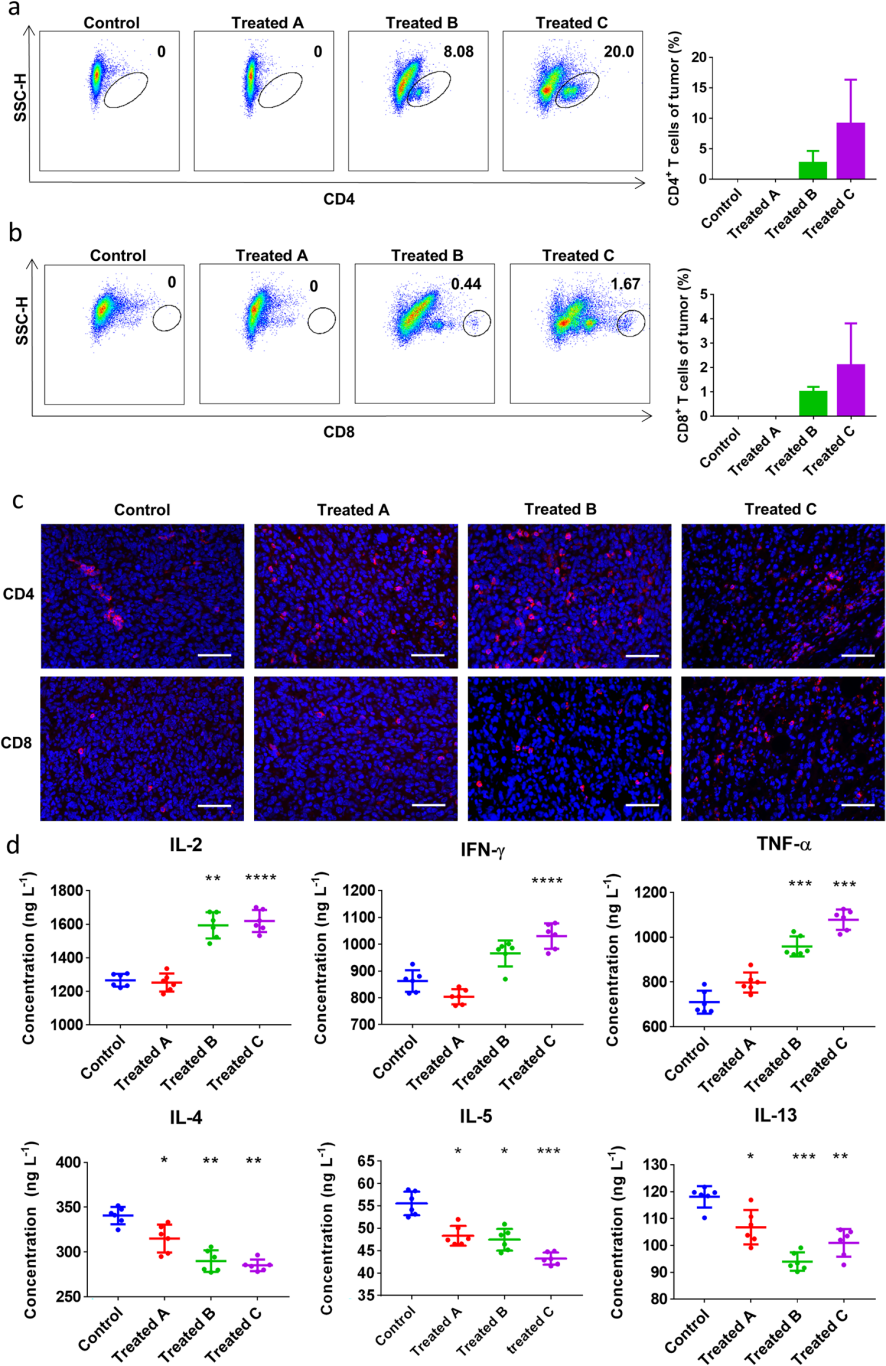
Feeding RCQ led to an increase in the proportion of CD4^+^ and CD8^+^ T cell in tumor environment, and induced a Th1 cells. Flow cytometric analysis for CD4^+^ (**a**) and CD8^+^ (**b**) T lymphocyte populations and proportion in tumor microenvironment (n = 4 mice). (**c**) Immunofluorescence staining of CD4^+^ (red), CD8^+^ (red), and nucleus (DAPI, blue), scale bar: 50 μm. (**d**) The inflammatory factors of IL-2, IFN-γ, TNF-α, IL-4, IL-5, and IL-13 in the tumors were detected by ELISA (n = 6 mice). Error bars represent mean ± SD. *p < 0.05, **p < 0.01, ***p < 0.001, ****p < 0.0001 vs control.

The above results have shown that RCQ enhanced antitumor efficacy. As reported previously that in the tumor microenvironment, CD8^+^ cytotoxic T lymphocytes, type 1 helper CD4^+^ T cells (Th1), M1 TAMs, and N1 cells have anti-tumor activity, while T regulatory (Treg) cells, type 2 helper CD4^+^ T cells (Th2), N2 cells and M2 TAMs known for their immunosuppressive and pro-tumor activity^38,39^. To clarify the effect of combination treatment on tumor immune microenvironment, we analyzed T cell content in the tumor microenvironment firstly and found that eating RCQ could increase T cell immune infiltration (Fig. 5). T cells were the main immune infiltrating cells, especially in the tumors of mice with long-term ingestion habits. Cytotoxicity CD8^+^ T lymphocytes are an essential part of tumor-specific cell adaptive immunity, which can specifically recognize and kill tumor cells. Moreover, the accumulation of CD4^+^ T cells will also promote the accumulation of CD8^+^ T cells, and the secreted IFN-γ (Interferon γ) will also increase the toxicity of CD8^+^ T cells^36,37^. It can be seen from Fig. 5a,b that the proportion of CD4^+^ and CD8^+^ T cells increased in treatment groups. In the treatment group, the proportion and absolute number of CD4^+^ and CD8^+^ T cells increased with the increased time of polyphenols intake which means as the time of taking RCQ increased, CD4^+^ and CD8^+^ T cells were recruited inside the tumor (Fig. 5c). Secondly, we tested the cytokines secreted by Th1/Th2 cells. Th1 cells mainly secrete IL(Interleukin)-2, IFN-γ, TNF (tumor necrosis factor)-α, etc., which mediate immune responses related to cytotoxicity and local inflammation. Th2 cells mainly secrete IL-4, IL-5, IL-13, etc. Studies have shown that using Th1 type T cell therapy is more effective than Th2 type in fighting tumors. We found that eating RCQ not only recruited T cells into the tumor microenvironment but also increased the cytokines secreted by Th1-type cells (IL-2, IFN-γ, TNF-α) (Fig. 5d). IL-2 secretion increased by 25.79% and 27.89% in Treated B and C. IFN-γ secretion increased by 11.91% and 19.41% in Treated B and C. TNF-α secretion increased by 12.33%, 35.04% and 51.86% in treatment group A, B, and C, respectively. The cytokines secreted by Th2-type cells (IL-4, IL-5, IL-13) are relatively reduced accordingly. Importantly, the longer the RCQ was consumed, the greater the Th1/Th2 ratio. After treatment, the secretion of IL-4 in group A, B and C decreased by 7.53%, 14.89%, and 16.30% compared with the control group, respectively. The secretion of IL-5 in the three groups decreased by 12.96%, 14.63%, and 22.16% compared with the control group, respectively. The secretion of IL-13 in the three groups decreased by 9.59%, 20.41%, and 14.51% compared with the control group, respectively. It can be inferred that Th1 cells occupied the dominant position in the anti-tumor effect and guided the tumor to develop in the direction of anti-tumor. In summary, the content of CD4^+^ Th1 and CD8^+^ cells in the tumor microenvironment increased in mice of the treatment group, and cytokines secreted by Th1 cells increased. In contrast, cytokines secreted by Th2 cells decreased. Moreover, IFN-γ can antagonize the production of immunosuppressive cytokines such as TGF-β and IL-10 which can promote the development of otherwise highly immunogenic tumors, improves the establishment of an effective antitumor memory immune response, and thus controls ongoing tumor growth^46^. In conclusion, the increase of IFN-γ secretion helps to reduce the immunosuppressive tendency of the tumor microenvironment. This is consistent with the reduction of tumor volume and the increase of anti-tumor immune cell content in the tumor microenvironment after the consumption of RCQ in mice in this study. This result convinced us that the administration of RCQ could reshape and repolarize the phenotype of TILs ins the tumor microenvironment.

#### RCQ reduced neutrophil recruitment to the tumor internal microenvironment and converted TANs to the N1 antitumor orientation while converting TAMs to the M1 type

The change of tumor-associated neutrophils (TANs) is not systemic but due to the recruitment or persistent changes in the tumor, so only the percentage of neutrophils inside the tumor would be measured^47^. "N1" TANs increased cytotoxicity and decreased immunosuppression by producing TNF-α, ICAM-1 (intercellular cell adhesion molecule-1), ROS, and FAS (TNF receptor superfamily, member 6) and reducing arginase expression ability. In contrast, "N2" TANs support tumor expansion by expressing arginase, MMP-9, VEGF, and many chemokines (including CCL (monocyte chemoattractant protein)-2 and CCL-5)^48^. Moreover, TGF-β in the tumor biases TANs towards the N2 TANs, while inhibition of TGF-β signaling induces the anti-tumor N1 TANs^49^. Human malignancies frequently exhibit mutations in the TGF-β pathway, and overactivation of this system is linked to tumor growth by promoting angiogenesis and inhibiting the innate and adaptive antitumor immune responses^50^. In this work, tumor immunosuppressive microenvironment resulted in a considerable rise in TGF-production in tumor microenvironment of tumor-bearing mice. It can be assumed that RCQ can disrupt the tumor immunosuppressive microenvironment since it can decrease TGF- production in the tumor microenvironment.

**Figure 6:**
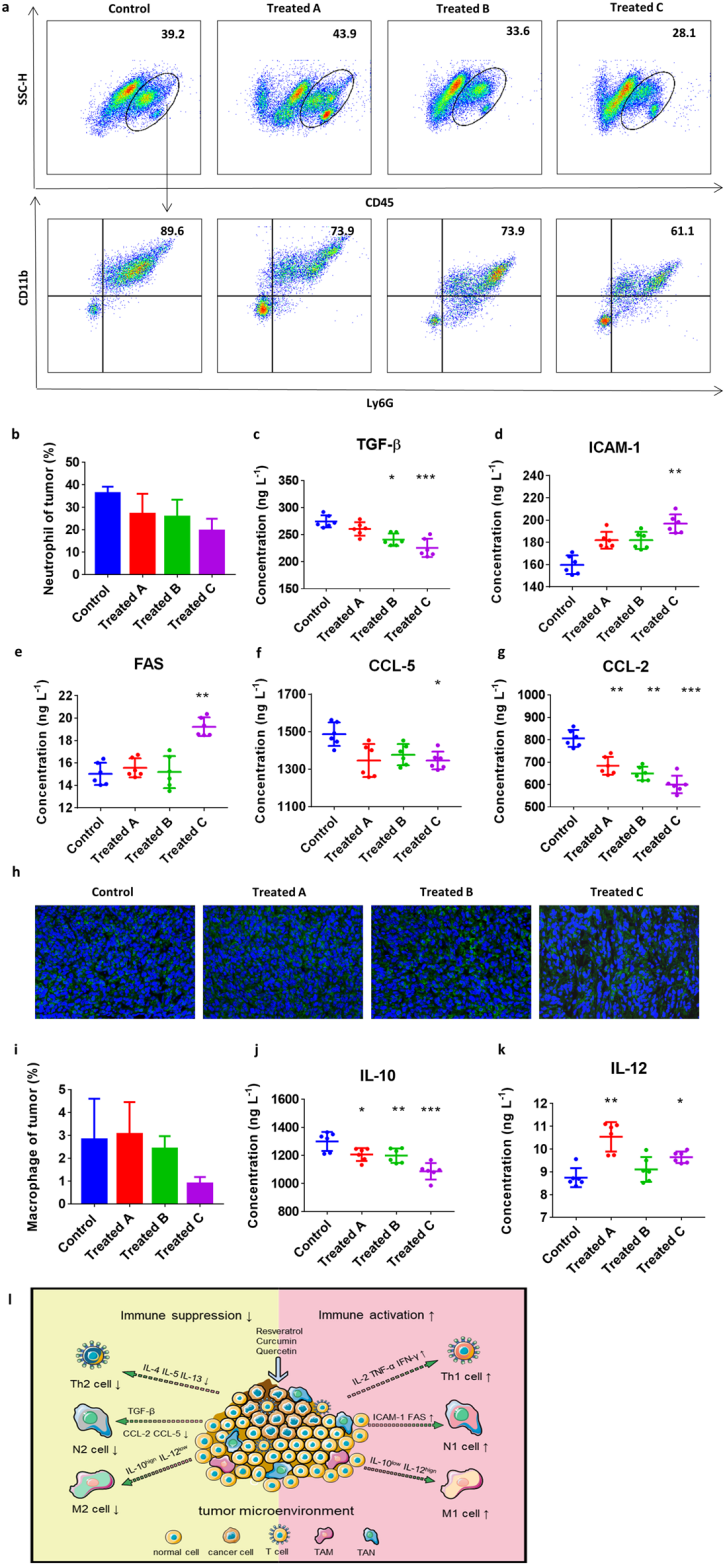
Feeding RCQ led to a decrease in the proportion of neutrophils (CD45^+^Ly6G^+^CD11b^+^) in the tumor microenvironment and induced the formation of N1 neutrophils and M1 macrophages. (**a**) Flow cytometry scatter dot plots for neutrophils. (**b**) Proportions of neutrophils in tumor microenvironment (n = 4 mice). The inflammatory factors of TGF-β (**c**), ICAM-1 (**d**), FAS (**e**), CCL-5 (**f**), and CCL-2 (**g**) in the tumor were detected by ELISA (n = 6 mice). (**h**) Immunohistochemical staining of macrophages (green) and nucleus (DAPI, blue). (**i**) Proportions of macrophages in tumor microenvironment (n = 4 mice). The inflammatory factors of IL-10 (**j**) and IL-12 (**k**) in the tumor were detected by ELISA (n = 6 mice). (**l**) Schematic of the antitumor mechanism of RCQ combinations on breast cancer-bearing mice. Error bars represent mean ± SD. *p < 0.05, **p < 0.01, ***p < 0.001, ****p < 0.0001 vs control.

Flow cytometry analyses were performed to examine levels of neutrophils in tumors of mice. According to previous literature, neutrophils were sorted with CD45^+^, Ly6G^+^, and CD11b^+^. As shown in Fig. 6a and Fig. S2, the consumption of RCQ reduced the recruitment of neutrophils. TANs in the treatment group decreased by 25.37%, 29.11% and 46.15%, respectively (Fig. 6b). The phenotype of TANs depended on the signals secreted by immune cells in the tumor microenvironment. We measured the level of neutrophils-associated chemokines-TGF-β (transforming growth factor-β), ICAM-1, CCL-2, CCL-5, FAS in the tumor homogenate to determine changes in the tumor immune microenvironment caused by the body's intake of RCQ. After taking RCQ, the proportion of neutrophil infiltration in the treatment group was decreased compared with the control group, and that of the treatment group C was still the lowest. By measuring the chemokine production, it can be concluded that the intake of RCQ would make TANs produce more N1 phenotype-related chemokines FAS, ICAM-1, and TNF-α, while reducing N2 TANs related chemokines TGF-β, CCL-2, and CCL-5. Compared with the control group, ICAM-1 increased by 23.07%, FAS increased by 27.91%, TGF-β decreased by 17.82%, CCL-5 decreased by 9.48%, and CCL-2 decreased by 25.51% in Treated C. In other treatment groups, we can also see the same trend of neutrophils-associated chemokines (Fig. 6c–g). This result not only indicated that RCQ promoted the development of antitumor N1 TANs but also reduced tumor neutrophil infiltration, which was consistent with H&E staining of tumors. N1 cells promote to induce CD8^+^ T cells recruitment and activation by producing chemokines (such as CCL-3, CXCL-9, and CXCL-10) and pro-inflammatory cytokines (such as IL-12, TNF-α, GM-CSF (granulocyte–macrophage colony stimulating factor), and VEGF (vascular endothelial growth factor))^51^. Therefore, combined with the increase of TNF-α and the recruitment of CD8^+^ T cells, it can also be inferred that the transformation of TANs to N1 type activated the anti-tumor activity of CD8, thus guiding the development of the tumor microenvironment toward the anti-tumor direction. Cancer recurrence was associated with lower T cells and higher neutrophils^52^, which confirmed that RCQ positively impacts tumor prognosis. It is instructive to explain these studies based on the difference in the activation state of neutrophils in tumors.

We found out a large number of TAMs in tissue immunostaining showed an overall downward trend, and group C decreased by 69.17% (Fig. 6h,i). We analyzed TAMs according to their polarization characteristics again. It was speculated that taking RCQ could still improve the polarization of M2 (high IL-10 and low IL-12). As can be seen from Fig. 6j–k, the changing trend was still the most obvious in group C, in which IL-10 decreased by 16.37% and IL-12 increased by 10.21%. This change showed the trend of low IL-10 and high IL-12; thus, we inferred that the polarization degree and immunosuppression of macrophages were improved.

M1 macrophages with high IL-12 and low IL-10 secretion characteristics are generally considered tumor-killing macrophages, mainly for anti-tumor and immune promotion. In contrast, M2 macrophages with low IL-12 and high IL-10 secretion characteristics promote tumor growth and metastasis and are related to poor tumor prognosis. In addition, we pay attention to the interaction of TILs subpopulation polarization. M1 TAMs are part of the Th1 polarization response, which mediates resistance to intracellular pathogens and tumors and triggers a tissue destruction response^53^. M1 TAMs drive the polarization and recruitment of Th1 cells through the expression of their cytokines and chemokines, thereby amplifying the Th1 response^54^. Similarly, CCL-2 and CCL-5 produced by tumors enhance the TAMs infiltration, resulting in the production of growth factors that promote tumor cell proliferation^55,56^. In our study, TAMs presented high IL-10 and low IL-12 trends in the treatment group, while the cytokines secreted by Th1 and N1 cells increased. For the treatment group, TAMs in the tumor immune microenvironment decreased and is converted to M1 TAMs, which was accompanied by a decrease for CCL-5 and CCL-2 and an increase for Th1 cells. Likewise, studies by Sumit Mukherjee et al*.* have found that the use of natural polyphenol combinations can repolarize macrophages in the tumor microenvironment, suggesting M1 TAMs transformation through decreased IL-10 secretion and increased IL-12 secretion^55^.

Summarily, this research demonstrated that the antitumor polyphenol combination enlarged the damage to tumor growth and remodeled the tumor immune microenvironment (Fig. 6l). Significantly, it confirmed from the side that there was an interaction among tumor immune infiltrating cells, which precisely strengthened the positive or negative tendencies of the tumor immune microenvironment. Especially, the longer mice in the treatment group were fed with RCQ, the larger the prevention function was. In the study of I. W. Cheuk, resveratrol enhanced chemosensitivity by reversing macrophage polarization in breast cancer^57^. Quercetin can inhibit the autophagy of M2-TAMs and induce their differentiation to M1-TAMs, thus inhibiting the proliferation of colorectal cancer cells^58^. Studies by Jincheng Wang et al. have found that curcumin is synergistic with cisplatin in the treatment of osteosarcoma by inhibiting M2-like polarization of tumor-associated macrophages. These studies potentially demonstrate the ability of RCQ to reverse immune cell polarity, which is consistent with our findings.

Our results revealed that positive phenotypic changes of TILs guide to break of immunosuppression and enhance tumor inhibition. It is a pity that our subject was based on mouse breast cancer cells, and the results should be confirmed in a larger study. Although our study still has limitations, such as the long duration of administration and failure to study accurate changes from tumorigenesis to advanced cancer, we still provide another evaluation way of the tumor prevention benefits of natural foods and a meaningful study of the inflammatory states in the tumor immune microenvironment. Another aspect that would be worth developing is to combine RCQ with traditional therapy, such as chemotherapy, radiotherapy, and rehabilitation. Above all, combination treatment is a promising work worth exploring by researchers.

In addition to reversing the immunosuppressive state of the tumor microenvironment, these natural antitumor components can specifically target some cancer-promoting cytokines and act on cancer-promoting molecular pathways. Resveratrol acts as an inhibitor that blocks all stages of carcinogenesis mediated by overexpression of growth factors and receptor tyrosine kinases. In particular, resveratrol has an effect on epidermal growth factor (EGF), which inhibits the occurrence, promotion, and progression of colorectal cancer, and simultaneously decreases the expression of vascular endothelial growth factor (VEGF) and promotes the activity of endothelial NO synthase (e-NOS). It prevents the formation of more aggressive tumor phenotypes, reduces the risk of new angiogenesis and metastasis, and hypoxia in cancer-related tissues^59,60^. Curcumin could suppress proliferation by attenuating the cell cycle via inhibiting the Wnt/β-catenin pathway, increasing the levels of p53, p21, and p27, and then inhibiting the levels of CDK4 and Cyclin D1^61^. Quercetin's anti-cancer effects have been studied in many studies, Quercetin is mediated by cellular signaling pathways such as Wnt/beta-catenin signaling pathway, phosphoinositol 3-kinase (PI3K)/protein kinase B (AKT) signaling pathway, Janus kinase (JAK)/signal transduction and transcriptional activator (STAT) signaling pathway, mitogen-activated protein kinase (MAPK) signaling pathway, etc. Its role in preventing tumor growth, proliferation, and progression^62^. The plant derivative is an appealing source of alternative anti-tumor drugs. The applied combination of flavonoids is a natural compound with potential anticancer effects in the adjuvant treatment of cancers.

Natural antitumor polyphenols have been used in human clinical trials at doses ranging from 300 to 2000 mg day^−1^, with different doses for different conditions. For example, eight patients were given curcumin orally at a dose of 375 mg/3 times/day for 6–22 months, and the final five patients completed the study, four of whom recovered completely and one had a complete reduction of swelling, but still had some movement limitations^63^. All patients had no side effects and no recurrence. Another clinical trial demonstrated that consuming polyphenols extended survival in men with prostate cancer at a dose^64^ of 4 mg day^−1^. Quercetin supplementation for 8 weeks at 500 mg day^−1^ significantly reduced inflammation, morning pain, and post-activity pain in women with rheumatoid arthritis^65^. Therefore, the recommended dose for humans is 0.3–4 mg day^−1^. Our study still has limitations, such as the long duration of administration and failure to study accurate changes from tumorigenesis to advanced cancer. And it failed to recommend appropriate human dosage; conduct animal studies to verify toxicology on a larger scale and over a longer period of ingestion; combine with other traditional anticancer drugs in the absence of a complete reduction of tumor volume.

## Conclusion

Long-term consumption of natural polyphenols can maximize the prevention and protection against cancer development. The combination of polyphenols was more effective than a single polyphenol, suggesting that a larger intake of fruits or vegetables was best for our daily fight against cancer development. In the process of cancer development, the intake of natural polyphenols can increase the ROS amount of cancer cells, reverse the development trend of cancer cells to malignant tumors, and delay the deterioration of tumors. By increasing T cell infiltration and reducing neutrophil infiltration, inflammatory factors and chemokines produced by T cell infiltration affected the polarization of infiltrating lymphocytes in the tumor, and promoted tumor anti-proliferation and sensitivity to immune cells. RCQ can make TILs avoid the characteristics of immune escape, enhance the immune sensitivity of the body, and promote the immune infiltrating cells in the tumor microenvironment to develop in the direction of anti-tumor. We provide another evaluation of the tumor prevention benefits of natural foods and a meaningful study of the inflammatory state of the tumor microenvironment. In the future, we hope to develop drugs or other therapies that combine these natural anticancer drugs to prevent or fight cancer.

## Methods

### Chemicals and reagents

Resveratrol, curcumin, and quercetin were purchased from MACKLIN (Shanghai Macklin Biochemical Co., Ltd), and the purity of all the natural products is ≥ 98%. These are dissolved in DMSO (60 mg mL^−1^ stock).

### Cell culture

4T1 cells (mouse breast cancer cell line) were ordered from the Chinese Academy of Sciences. Dulbecco’s modified Eagle’s medium (DMEM) and fetal bovine serum (FBS) were purchased from Gino Biological Pharmaceutical Technology Co., Ltd. (Hangzhou, China). Cells were maintained in a DMEM culture medium, supplemented with 10% FBS, and incubated at 37 °C in a 5% CO_2_ atmosphere.

### Cell viability assays

Cell viability assays were performed using a CCK-8 Kit (DOJINDO Laboratories, Japan), following the manufacturer's instructions. The cell viability was expressed as the percentage of viable cells within the total cells. 4T1 cells were seeded in 96-well plates (5 × 10^3^ cells per well), incubated for a 24 h attachment period, and cells were treated with differing concentrations of Res, Cur, Que, and RCQ for an additional 24 h. The cell viability of each well was determined with the CCK-8 kit following the instruction manual, and the 450 nm absorbance of each well was measured using a microplate reader (Thermo, USA).

### Annexin V-FITC/PI assays

4T1 cells were seeded and allowed to incubate overnight. In this experiment, 10^4^ 4T1 cells were spread in confocal dishes overnight and then incubated with 1 mL Res, Cur, Que, and RCQ (15 mg L^−1^) for 8 h at 37 °C with 5% CO_2_. The cells were stained with Annexin V-FITC and PI according to the protocol (DOJINDO Laboratories, Japan). Confocal microscopy was used to measure the fluorescence intensity of the cells (annexin V-FITC, λ_ex_ = 488 nm, λ_em_ = 500–560 nm; PI, λ_ex_ = 488 nm, λ_em_ = 600–680 nm).

### Reactive oxygen species (ROS) generation

ROS was measured using a Reactive Oxygen Species Assay Kit (Beyotime Laboratories, China). In this experiment, 10^4^ 4T1 cells were spread in confocal dishes overnight and then incubated with 1 mL Res, Cur, Que, and RCQ (15 mg L^−1^) for 8 h at 37 °C with 5% CO_2_. It used the oxidation-sensitive fluorophore 2′7′-dichlorofluorescein diacetate (DCFH-DA), a non-fluorescent compound, to freely pass through the cells and was hydrolyzed by esterases to 2′,7′-dichlorofluorescein (DCFH). ROS (green) production was determined by the conversion of 2′,7′-dichlorodihydrofluorescein diacetate (DCFH-DA) to 2′,7′-dichlorodihydrofluorescein. Briefly, cells with Res, Cur, Que, and RCQ were harvested and incubated with 20 μmol L^−1^ DCFH-DA dissolved in an FBS-free medium at 37 °C for 20 min and were then washed three times with an FBS-free medium. The fluorescence of DCF was measured via flow cytometry and laser scanning confocal microscope (FITC: λ_ex_ = 488 nm, λ_em_ = 525 nm) to determine the generation of ROS levels. Intracellular ROS production was expressed as fluorescence production (analyzed by Image J).

### Measurement of mitochondrial membrane potential (MMP)

In this experiment, 10^4^ 4T1 cells were spread in confocal dishes overnight and then incubated with 1 mL Res, Cur, Que, and RCQ (15 mg L^−1^) for 8 h at 37 °C with 5% CO_2_. The JC-1 probe was used to estimate changes in mitochondrial membrane potential. 4T1 cells were treated with 15 mg L^−1^ Res, Cur, Que, and RCQ for 2 h incubated with JC-1 staining solution (DOJINDO Laboratories, Japan) for 20 min and immediately observed under confocal microscopy (Thermo, USA). An excitation wavelength of 488 nm was selected for JC-1, and the emission wavelengths for JC-1 monomer and JC-1 aggregate were set to 515–545 nm and 570–600 nm, respectively. Red fluorescence represented the mitochondrial aggregate JC-1, and green fluorescence indicated the monomer JC-1. Photograph showing JC-1 red, JC-1 green and merge image. Loss of ΔΨm was demonstrated by the change in JC-1 fluorescence from red (JC-1 aggregates) to green (JC-1 monomers). The fluorescence of JC-1 monomer and JC-1 aggregate was measured via flow cytometry and laser scanning confocal microscope to determine the change of MMP levels.

### Animal subjects

All procedures were approved by the Ethics Committee of Shanghai University with Approval No. ECSHU 2021-119 and were conducted in strict accordance with the guidelines for animal care. Female BALBc mice (about 6–8 weeks old and a body weight of 20 ± 2 g) were purchased from the Shanghai Jiesijie Experimental Animal Center (Shanghai, China). All the animals were housed in plastic cages with a stainless-steel mesh lid in a ventilated animal room. Room temperature was maintained at 23 ± 2 °C and relative humidity at 60 ± 10%, and a 12 h light–dark cycle was used. Distilled water and sterilized food for the mice were available ad libitum. Before inoculation, the mice were acclimated to this environment for 7 days. The minimal possible number of animals was sacrificed while all efforts were made to reduce their suffering. The ARRIVE guidelines (Animal Research: Reporting of In Vivo Experiments) were followed to report animal experiments.

### Animal flank tumor models

Mice were injected on the right flank with 4T1 tumor cells in the appropriate syngeneic host. Following treatments as outlined below, tumor growth was followed with measurement once 2 days. All experiments had at least five mice per group and were repeated at least two times. When needed (i.e., for FACS, RNA, cell subsets isolation, and so forth), flank tumors were harvested from the mice, minced, and digested with 2 mg mL^−1^ DNase I (Solorbio, China) and 4 mg mL^−1^ collagenase type IV (Solorbio, China) at 37 °C for 1 h. And some of them are used for H&E staining.

### Animals treated breast cancer

BALBc mice were injected 2 × 10^5^ 4T1 cells subcutaneously. The treatment plan was divided into three types. The mice in the Treated A group were given RCQ by intragastric administration for 30 days after subcutaneous injection of 4T1 breast cancer cells. The mice in the Treated B group were consumed RCQ for 15 days for prevention, then injected subcutaneously with 4T1 breast cancer cells and continued to gavage RCQ for 30 days. The mice in the Treated C group were consumed RCQ for 30 days for prevention, then injected subcutaneously with 4T1 breast cancer cells, and continued to gavage RCQ for 30 days. Each mouse was fed 60 mg kg^−1^ orally five times a week, 200 μL each time. RCQ in PBS plus 1% DMSO was prepared by dilution from Res, Cur, and Que solutions in DMSO. The control group was fed with PBS plus 1% DMSO. From Monday through Friday, RCQ was given to the mice five times a week, once per day. In previous studies, the non-toxic dose of these antitumor polyphenols in tumor mice was generally between 5–140 mg kg^−1^. In this study, the dose of RCQ was 60 mg kg^−1^ (including 24 mg kg^−1^ Res, 24 mg kg^−1^ Cur, and 12 mg kg^−1^ Que), which was the non-toxic dose. Grapes contain the highest content of polyphenols, which can reach 50–100 mg kg^−1^. The recommended dosage for humans is 0.3–1 g per day, equivalent to 3 kg of grapes, but full amounts of polyphenols can be obtained by taking extract capsules. According to the body surface area method, the conversion factors between different animals and humans are different. The dose given to mice was about 12.3 times that given to humans. 60 mg kg^−1^ (including 24 mg kg^−1^ Res, 24 mg kg^−1^ Cur, and 12 mg kg^−1^ Que) translated into an edible human dose of 738 mg/day (including 295.2 mg Res, 295.2 mg Cur, and 147.6 mg Que). On the other hand, applying allometric principles and taking different caloric demands of mice and humans into account^66–68^, it is possible that, related to body weight, humans need an approximately 5–10-fold lower dose of RCQ than mice for an effect. Therefore, the intake of RCQ for humans can be reduced as appropriate.

### Flow cytometric analysis of tumors

Tumor cells were studied by FACS analysis as previously described. The following fluorescently labeled antibodies were purchased from BD Bioscience: CD3-FITC, CD4-Percp-5.5, and CD8-PE. CD45-Percp-5.5, CD11b-APC, Ly6G-FITC. And F4/80-PE are from Biolegend (CA, USA). All flow cytometry was done using a Beckman Coulter flow cytometer (San Jose, CA). Data analysis was done using FlowJo software (Ashland, OR.)

### Immunohistochemical staining of tumors

Animals bearing tumors were euthanized, and the tumors were immediately placed in Tissue-Tek OCT compound (Sakura Finetek USA, Inc., Torrance, CA) to be stored at − 80 °C, followed by Sectioning and staining. Monoclonal antibodies against T cells (anti-CD4^+^ and CD8^+^), macrophages (anti-F4/80), and Ly6G^+^ cells (anti-Ly6G^+^) were obtained from BD Biosciences.

### Cytokine production in vitro

The tumors were ground into a tissue suspension. Cytokine levels of IL-2, IL-4, IL-5, IL-13, IL-12, TNF-α, IL-10 and TGF-β, IFN-γ, CCL-2, CCL-5, ICAM-1, FAS were measured by ELISA (Shanghai Hu Ding Biological Technology Co, Ltd).

### RT-qPCR

RT-qPCR was performed to determine the change in the mRNA expression levels of Bax, Bcl-2, caspase-3, and caspase-9 genes after various treatments. 15 mg L^−1^ Res/Cur/Que/RCQ was added to 6-well plates, which is overwritten by 1.5 × 10^5^ 4T1 cells for 10 h. All RT-qPCR tests were performed using standard laboratory procedures. Table 1 represents the primer sequences of the mentioned genes as well as the GAPDH gene which was used as the internal control.

**Table 1:** Primer sequences used for real-time PCR.

	Forward primer (5′–3′)	Reverse primer (5′–3′)
Bax	TTGCTACAGGGTTTCATCCAGG	GCAAAGTAGAAGAGGGCAACCA
Bcl-2	CTACCGTCGTGACTTCGCAGA	ACACATGACCCCACCGAAC
Caspase3	TGGAAAGCCGAAACTCTTCATCA	CCACGACCCGTCCTTTGAAT
Caspase9	GCCACTGCCTCATCATCAACA	TTTCTTGGCAGTCAGGTCGTT

### Western blot

Western Blot was performed to determine the change in the protein expression levels of Bax, Bcl-2, caspase-3, and caspase-9 genes after various treatments. 15 mg L^−1^ Res/Cur/Que/RCQ was added to 6-well plates, which is overwritten by 4T1 cells for 10 h. All western blot tests were performed using standard laboratory procedures including sample preparation, protein concentration determination, denaturing the protein, SDS-PAGE, transferring the protein from the gel to the membrane, antibody staining, chemiluminescence, and image analysis.

## Supplementary Information

Supplementary Figures.

## Data Availability

The datasets used and/or analyzed during the current study are available from the corresponding author on reasonable request.
