# Correction: The effect of resveratrol, curcumin and quercetin combination on immuno-suppression of tumor microenvironment for breast tumor-bearing mice

**DOI:** 10.1038/s41598-025-04321-9

**Published:** 2025-06-06

**Authors:** Chenchen Li, Yajun Xu, Junfeng Zhang, Yuxi Zhang, Wen He, Jiale Ju, Yinghua Wu, Yanli Wang

**Affiliations:** 1 School of Medicine and School of Environmental and Chemical Engineering, Shanghai University, Shanghai, 200444 People’s Republic of China; 2 Key Laboratory of Tropical Translational Medicine of Ministry of Education, International Associated Research Center for Intelligent Human Computer Collaboration on Tumor Precision Medicine, School of Pharmacy and The First Affiliated Hospital, Hainan Medical University, Haikou, 571199 Hainan China

Correction to: *Scientific Reports* 10.1038/s41598-023-39279-z, published online 16 August 2023

The original version of this Article contained an error in Fig. 5, where panel CD4 in the Treated B group in Fig. 5C was duplicated in panel CD8 in the same group. The original Fig. 5 and accompanying legend appear below.

**Fig. 5:**
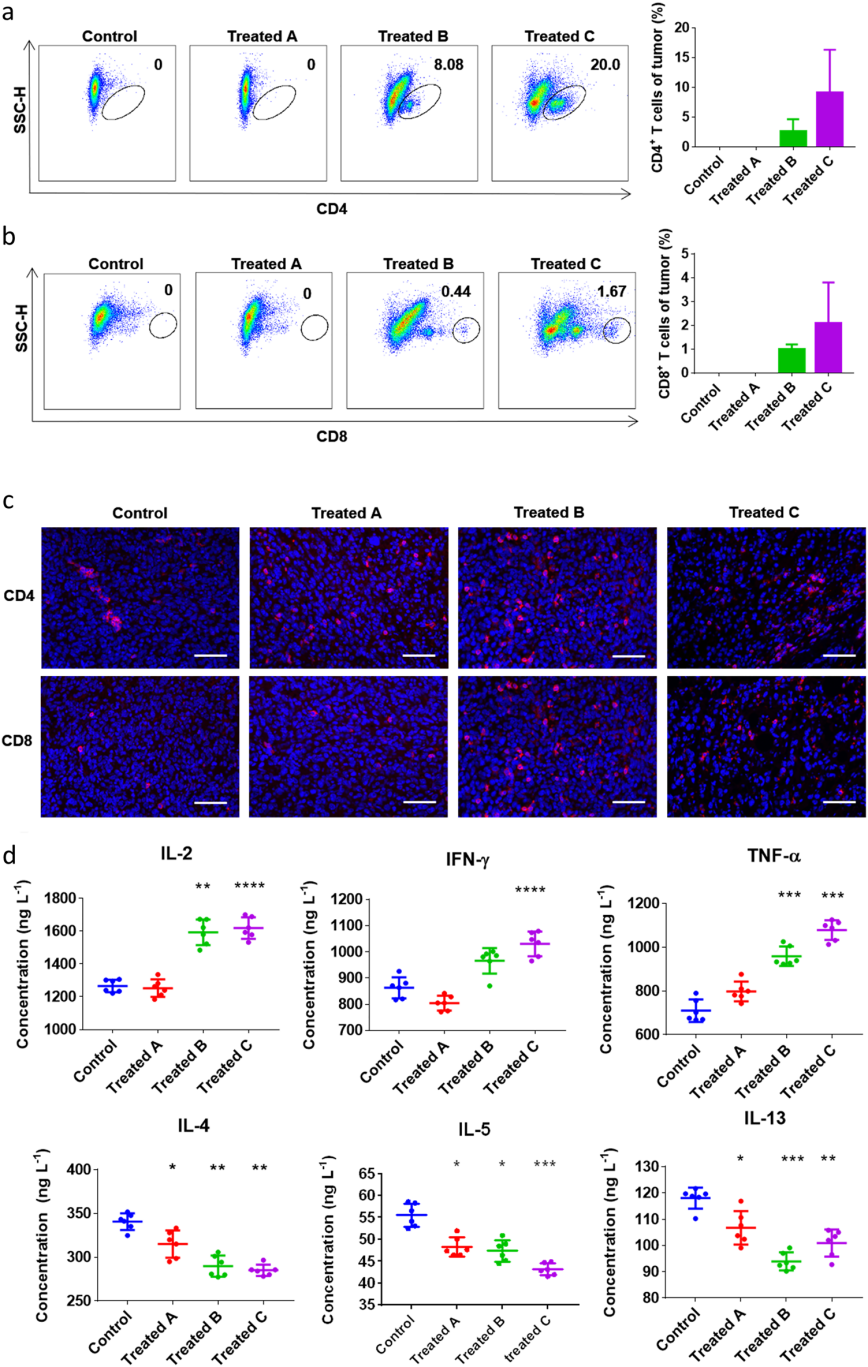
Feeding RCQ led to an increase in the proportion of CD4^+^ and CD8^+^ T cell in tumor environment, and induced a Th1 cells. Flow cytometric analysis for CD4^+^ (**a**) and CD8^+^ (**b**) T lymphocyte populations and proportion in tumor microenvironment (n = 4 mice). (**c**) Immunofluorescence staining of CD4^+^ (red), CD8^+^ (red), and nucleus (DAPI, blue), scale bar: 50 μm. (**d**) The inflammatory factors of IL-2, IFN-γ, TNF-α, IL-4, IL-5, and IL-13 in the tumors were detected by ELISA (n = 6 mice). Error bars represent mean ± SD. *p < 0.05, **p < 0.01, ***p < 0.001, ****p < 0.0001 vs control.

The original Article has been corrected.

