# Supplementary material for: The effect of resveratrol, curcumin and quercetin combination on immuno-suppression of tumor microenvironment for breast tumor-bearing mice
Source: Sci Rep. 2023 Aug 16;13:13278. doi: 10.1038/s41598-023-39279-z (PMC10432483; doi:10.1038/s41598-023-39279-z)
Supplement: Supplementary file 1 — Supplementary Figures. [file 41598_2023_39279_MOESM1_ESM.docx]

Supplementary Information

**The Effect of Resveratrol, Curcumin and Quercetin Combination on Immuno-suppression of Tumor Microenvironment for Breast Tumor-bearing Mice**

**Chenchen Li** 1,2,+**, Yajun Xu** 1,+**, Junfeng Zhang**1,+**, Yuxi Zhang**1**, Wen He**1**, Jiale Ju**1**, Yinghua Wu**1 **and Yanli Wang**1 *

1School of Medicine & School of Environmental and Chemical Engineering, Shanghai University, Shanghai 200444, P. R. China

2Key Laboratory of Tropical Translational Medicine of Ministry of Education, International Associated Research Center for Intelligent Human Computer Collaboration on Tumor Precision Medicine, School of Pharmacy, Hainan Medical University, Haikou 571199, Hainan, China.

*corresponding. wangyanli@staff.shu.edu.cn

+these authors contributed equally to this work


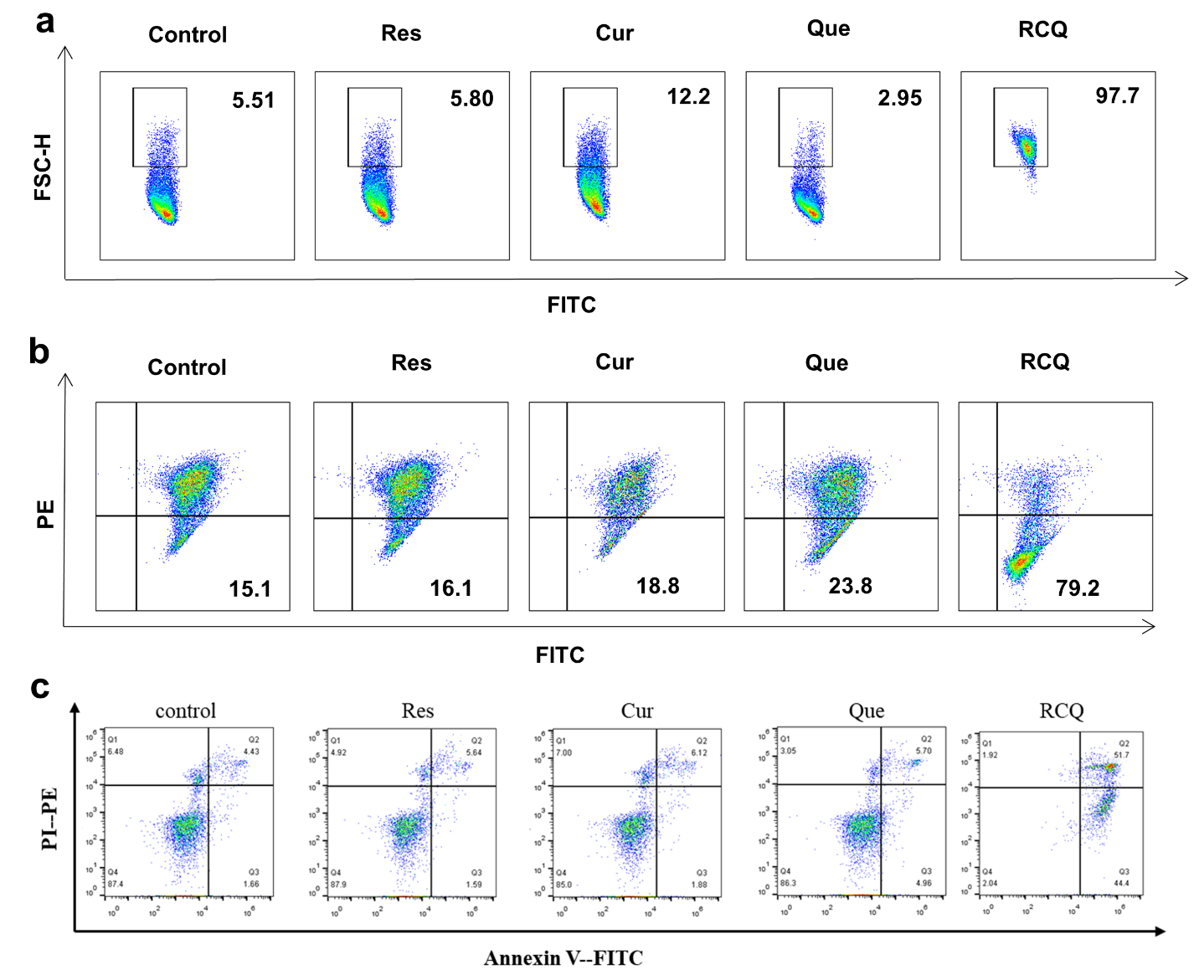


**Figure S1.** Flow cytometry analysis of (a) ROS production in 4T1 cells by 15 mg L^-1^ Res/Cur/Que/RCQ after 2 h, respectively; (b) Mitochondrial membrane potential (Δ Ψm) in 4T1 cells incubated with 15 mg L^-1^ Res/Cur/Que/RCQ after 8 h respectively; (c) annexin V/PI staining of 4T1 cells after incubated with RCQ 8 h.


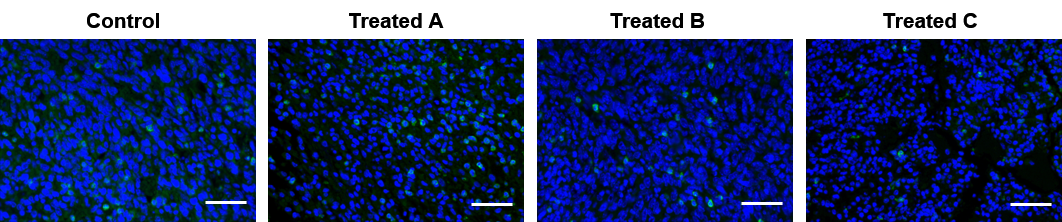


**Figure S2.** Immunofluorescence staining of Ly6G^+^ (green) and nucleus (DAPI); scale bar, 50 μm.


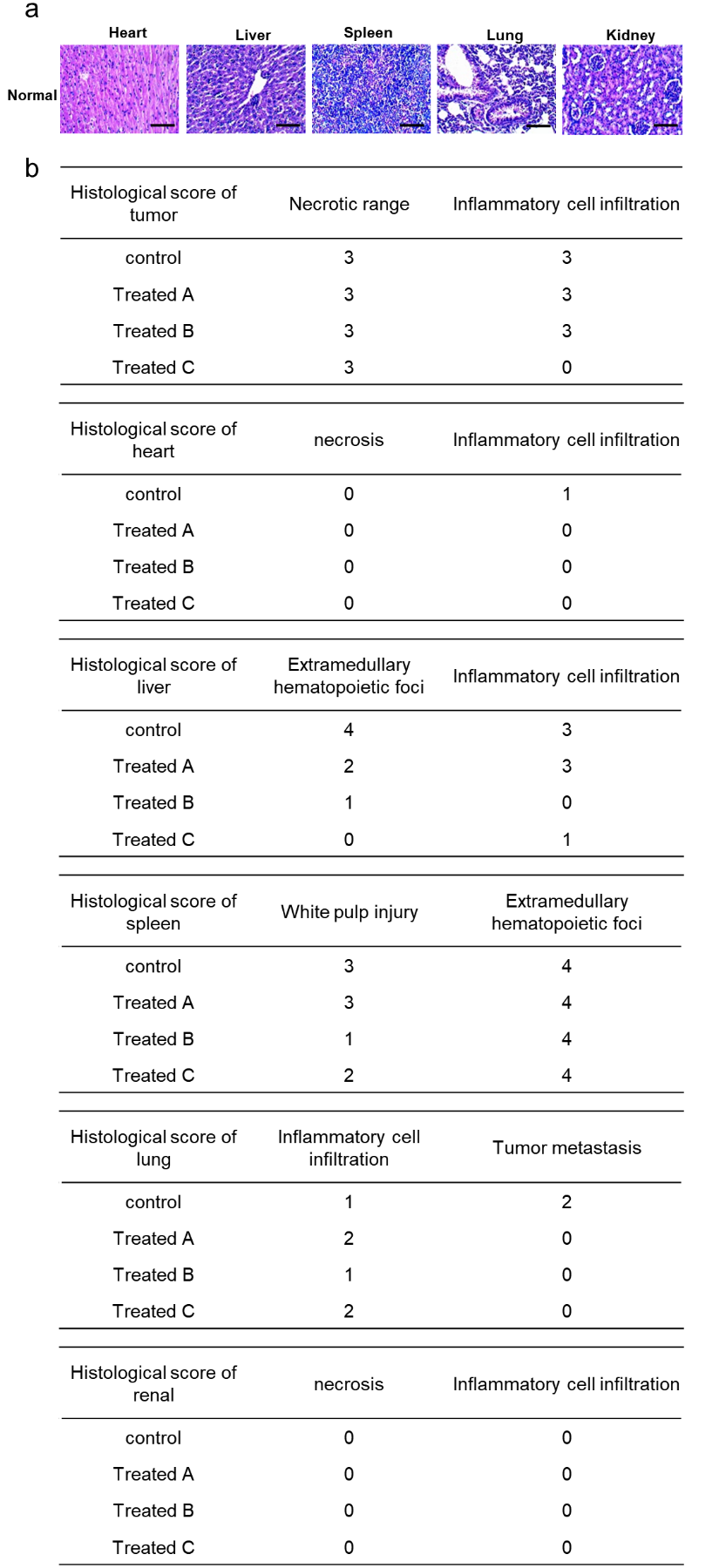


**Figure S3.** (a) HE staining of viscera of normal mice (without tumor); scale bar, 100 μm. (b) the histology score of tumor mice.


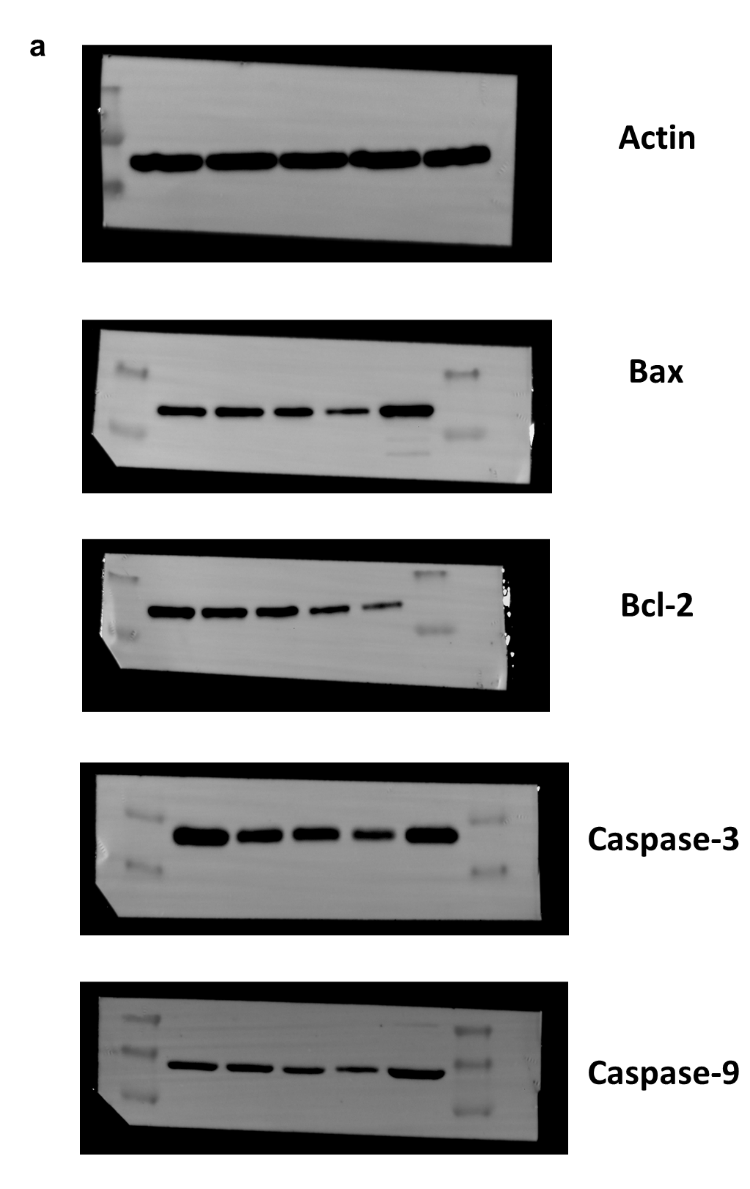


**Figure S4.** (a)The protein expression levels of Bax/Bcl-2/caspase-9/caspase-3 by Western Blot.
